# Transcranial electrical stimulation during functional magnetic resonance imaging in patients with genetic generalized epilepsy: a pilot and feasibility study

**DOI:** 10.3389/fnins.2024.1354523

**Published:** 2024-03-19

**Authors:** Zachary Cohen, Mirja Steinbrenner, Rory J. Piper, Chayanin Tangwiriyasakul, Mark P. Richardson, David J. Sharp, Ines R. Violante, David W. Carmichael

**Affiliations:** ^1^Department of Biomedical Engineering, School of Biomedical Engineering and Imaging Sciences, King's College London, London, United Kingdom; ^2^Department of Neurology, Charité – Universitätsmedizin Berlin, Berlin, Germany; ^3^University College London Great Ormond Street Institute of Child Health, University College London, London, United Kingdom; ^4^Department of Basic and Clinical Neuroscience, Institute of Psychiatry, Psychology, and Neuroscience, King’s College London, London, United Kingdom; ^5^The Computational, Cognitive and Clinical Neuroimaging Laboratory, Department of Medicine, Imperial College London, London, United Kingdom; ^6^School of Psychology, Faculty of Health and Medical Sciences, University of Surrey, Guildford, United Kingdom

**Keywords:** epilepsy, Juvenile Myoclonic Epilepsy, transcranial electrical stimulation, functional MRI, neuromodulation, sensorimotor

## Abstract

**Objective:**

A third of patients with epilepsy continue to have seizures despite receiving adequate antiseizure medication. Transcranial direct current stimulation (tDCS) might be a viable adjunct treatment option, having been shown to reduce epileptic seizures in patients with focal epilepsy. Evidence for the use of tDCS in genetic generalized epilepsy (GGE) is scarce. We aimed to establish the feasibility of applying tDCS during fMRI in patients with GGE to study the acute neuromodulatory effects of tDCS, particularly on sensorimotor network activity.

**Methods:**

Seven healthy controls and three patients with GGE received tDCS with simultaneous fMRI acquisition while watching a movie. Three tDCS conditions were applied: anodal, cathodal and sham. Periods of 60 s without stimulation were applied between each stimulation condition. Changes in sensorimotor cortex connectivity were evaluated by calculating the mean degree centrality across eight nodes of the sensorimotor cortex defined by the Automated Anatomical Labeling atlas (primary motor cortex (precentral left and right), supplementary motor area (left and right), mid-cingulum (left and right), postcentral gyrus (left and right)), across each of the conditions, for each participant.

**Results:**

Simultaneous tDCS-fMRI was well tolerated in both healthy controls and patients without adverse effects. Anodal and cathodal stimulation reduced mean degree centrality of the sensorimotor network (Friedman’s ANOVA with Dunn’s multiple comparisons test; adjusted *p* = 0.02 and *p* = 0.03 respectively). Mean degree connectivity of the sensorimotor network during the sham condition was not different to the rest condition (adjusted *p* = 0.94).

**Conclusion:**

Applying tDCS during fMRI was shown to be feasible and safe in a small group of patients with GGE. Anodal and cathodal stimulation caused a significant reduction in network connectivity of the sensorimotor cortex across participants. This initial research supports the feasibility of using fMRI to guide and understand network modulation by tDCS that might facilitate its clinical application in GGE in the future.

## Introduction

Epilepsy is a neurological disorder that affects approximately 70 million people worldwide and is common in both children and adults (Ngugi et al., 2010). Despite the availability of anti-seizure medications, around one-third of patients have seizures that cannot be adequately controlled by medication alone. Recent drug development has not yielded many new solutions, with the rate of drug-resistant epilepsy remaining relatively stable for the past 30 years (Brodie, 2017). Even in cases where anti-seizure medication is effective, up to 17% of individuals experience limiting side effects from the medication (Chen et al., 2017). While epilepsy surgery is a good option for some patients with focal epilepsy, a third continue to have seizures despite surgery and it is generally not an option for those with genetic generalized epilepsy (GGE) (Baud et al., 2018). As a result, a significant portion of patients, particularly those with GGE, are left without effective treatment. Novel, and preferably non-invasive treatments, are urgently needed.

Transcranial electrical stimulation (tES) is a promising novel therapeutic approach for drug-resistant epilepsy (Yang et al., 2020; Simula et al., 2022). tES involves the application of a low-intensity electric current (typically <2 mA) to the brain via scalp electrodes. tES can be delivered using different waveforms, the most common being: (1) Direct current stimulation (tDCS), which is applied with a uniform, unidirectional current flowing from the anode to the cathode. While being an over-simplification, from results obtained from motor cortex stimulation, if the region of interest is under the anode (i.e., during anodal tDCS) it is broadly believed that this will result in a local increase of neuronal activity. Conversely, if it is under the cathode (i.e., during cathodal tDCS) it will lead to a decrease in neuronal activity (Bestmann and Walsh, 2017). Sham stimulation, in which the current is ramped up at the same rate as tDCS but then quickly turned off, is typically used as the control condition in investigations (Bestmann and Walsh, 2017).

Previous pre-clinical and clinical studies have shown that tDCS can be effective in reducing interictal epileptiform discharges (IEDs) and seizures in individuals affected by epilepsy (San-Juan et al., 2017; Simula et al., 2022). A recent systematic review of the use of tDCS in epilepsy demonstrated that tDCS in epilepsy is safe and led to a relevant seizure reduction in most clinical studies, though results varied greatly due to different stimulation paradigms (Simula et al., 2022). So far there exists only one double-blind, randomized, sham-controlled trial and almost all studies have been done in patients with focal epilepsy (Yang et al., 2020). Data on the application of tES in GGE is very limited, and to date it has been found to be ineffective (San-Juan et al., 2016). A recent meta-analysis of clinically established neurostimulation techniques such as vagus nerve and deep brain stimulation has shown a significant effect on seizure frequency in GGE (Haneef and Skrehot, 2023). While the underlying mechanisms of these techniques differ, they provide encouraging evidence to further investigate the use of tES in GGE. Current evidence from *in-vitro* and human studies assessing functional connectivity and using computational models indicate that the effects of tES are mainly achieved through the modulation of large brain networks, instead of focal brain activity (Simula et al., 2022). One possible target in patients with GGE may be the sensorimotor network, which has been shown to have greater network synchrony in the minute before epileptiform discharge onset (Tangwiriyasakul et al., 2018), in comparison to their healthy relatives (Tangwiriyasakul et al., 2019). This network has also been a frequent target of tES in studies outside of epilepsy, which provide existing protocols to build from (Violante et al., 2017; Mencarelli et al., 2020).

The primary objective of this study was to establish the feasibility and safety of using tDCS during fMRI in both healthy participants and patients with Juvenile Myoclonic Epilepsy (JME), a subtype of GGE (Hirsch et al., 2022). The secondary objective was to investigate the acute changes in brain connectivity within the sensorimotor network in both groups. Our hypotheses were that tES would (a) be low-risk and tolerable in both groups and (b) lead to altered connectivity in the sensorimotor network. To test these hypotheses, we applied an established protocol and analyzed network connectivity using measures of degree centrality to determine if network modulation might be feasibly measured via this approach.

## Materials and methods

### Participants

Seven healthy control participants were recruited via email adverts. One healthy control was later excluded due to diagnosis of a neurological disease while the study was ongoing. Ethical approval to study our healthy participants was granted through the local ethics boards Research Ethics Committee (London – West London and GTAC). Three patients with JME being treated at King’s College Hospital were recruited. Ethical approval to study our patient group was granted by the Health Research Authority and Health and Care Research Wales (HCRW): REC reference: 19/LO/1668. All participants signed an informed consent form.

### Transcranial electrical stimulation

**Figure 1:**
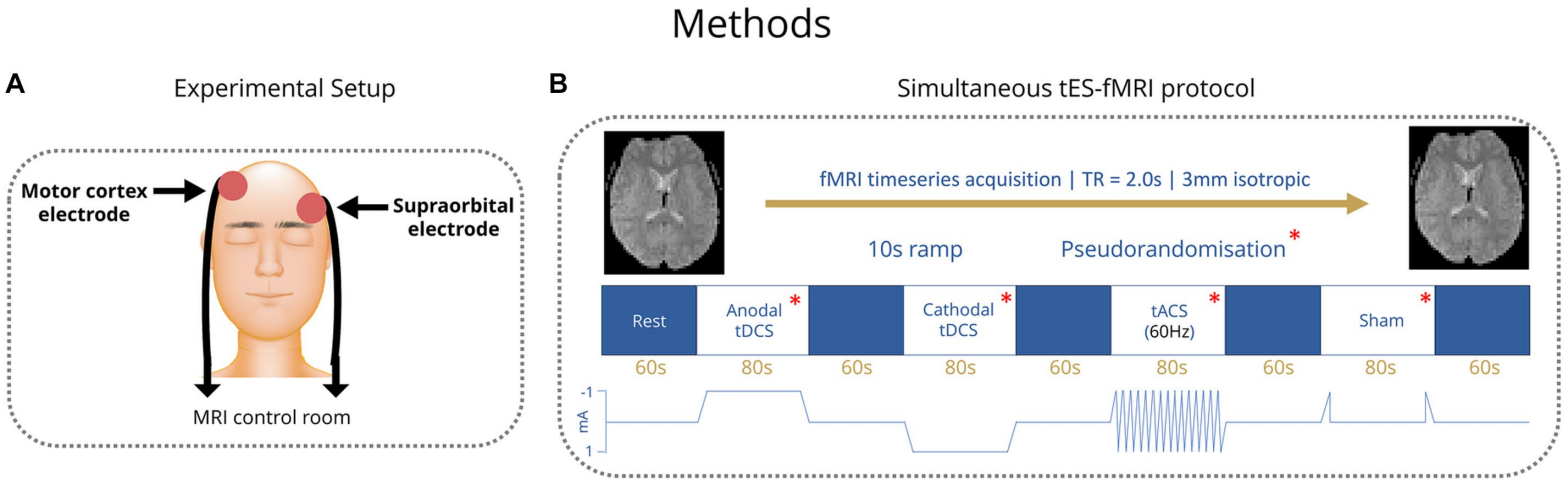
**(A)** Placement of tES electrodes over the right motor cortex and the left supraorbital region. **(B)** TES stimulation paradigm blocks. Order of conditions pseudorandomized.

All participants received transcranial electric stimulation (tES) from MR-conditional battery-driven stimulators (NeuroConn GmbH, Ilmenau, Germany). Electrode positions were marked on the scalp using an EEG cap. Stimulation electrodes were placed over the right motor cortex, with the middle of the electrode positioned over FC6, and the left supraorbital region with the middle of the electrode positioned over AF7 (Figure 1A) (Wolf and Goosses, 1986). Electrodes were rectangular 5x7cm and placed on the participant’s heads using an evenly spread conductive paste, approximately half a centimeter in thickness, the exact amount was not measured (Ten20, D.O. Weaver, Aurora, CO, USA). The tES setup was in place throughout the MRI session (including structural imaging). Impedances were kept below 10 kΩ and checked in each individual before they went into the scanner and again before starting stimulation. Participants were first exposed to short blocks of stimulation (with current increasing from 0.2 mA to 1 mA) before entering the scanner to ensure they were comfortable with it. Overall, we followed the hardware arrangement as previously described by Violante et al. (2017): In summary, the stimulators were placed outside the shielded MR room. The current from the stimulators was delivered into the scanner room after being filtered from RF noise by two filter boxes, one placed in the operator room and another inside the scanner bore connected via a waveguide. The second box was connected to the stimulation electrodes via MR-conditional cables. The wire routing pattern was out the back of the bore and around the control room, wires were connected to the patient shortly before the scan and positioned as straight as possible to not create loops. The filter box and wires were secured with tape. The stimulator was controlled and monitored using an in-house written Matlab code (by IRV) via a NI USB-6216 BNC data acquisition unit (National Instruments, Austin, USA). The beginning and end of each stimulation block was controlled via an external trigger sent to the NI USB-6216 BNC from the computer running the experimental paradigm (which received TTL triggers from the MR scanner). The setup used to route stimulation through the participant inside the scanner did not introduce artifacts in the fMRI signal (Li et al., 2019; Violante et al., 2023).

In healthy control subjects, four different tES conditions were applied: Anodal transcranial direct current stimulation (tDCS), cathodal tDCS, transcranial alternating current stimulation (tACS) with 60 Hz and a sham condition, where current was ramped up to test levels and then stopped. Anodal and cathodal tDCS were applied with 1 mA current intensity and tACS 1 mA peak to peak. Conditions were applied in blocks of 80 s with 60 s rest periods between them (Figure 1B). Healthy control participants completed four runs of these four conditions. The order of the conditions within each run was pseudorandomized to allow trends to be measured irrespective of the order of conditions. After the scan, healthy controls were asked to fill in a short form about the effects they experienced during tES. Patients received the same number of conditions within each run but with the tACS condition replaced by another condition (sham, anodal tDCS or cathodal tDCS) that was altered in each run such that over four runs, conditions were balanced. Stimulation parameters (i.e current intensity and montage) in patients matched those of controls. The tACS condition was found to commonly elicit a flickering visual disturbance (phosphenes) in the healthy control group from the survey. Among patients with genetic generalized epilepsies and especially in those with JME, there is a reported high prevalence of photosensitivity of up to 30.5%, which means that flickering lights can elicit seizures in those individuals (Wolf and Goosses, 1986; Fisher et al., 2005). Therefore, in the patient group, the tACS condition was removed due to potential health risks that could be associated with seizure induction.

### Functional magnetic resonance imaging (fMRI)

#### Image acquisition

Three hundred and sixteen echo-planar images were acquired per run. Healthy participants were scanned on a Siemens Verio 3 T at the Clinical Imaging Facility at Imperial College London (3 mm isotropic voxels, repetition time [TR] = 2 s, echo time = 30 ms, flip angle 80°). Patients were scanned on GE 3 T at King’s College London (3 mm isotropic voxels, repetition time [TR] = 2 s, echo time = 30 ms, flip angle 80°). During scanning we applied a movie paradigm, participants watched a cartoon (Gulliver’s Travels), chosen to better control attention levels, preventing them from falling asleep. This approach was selected because isolated brain state dynamics in fMRI using a movie paradigm could be more reliably attributed to a disease state or progression change (van der Meer et al., 2020).

#### Image pre-processing

Pre-processing of fMRI data was performed with Statistical Parametric Mapping (SPM 12) using MATLAB (R2021a; MathWorks). The first five volumes of each fMRI run were removed to account for T1-related signal fluctuations. Following realignment to correct for head motion across each run, the Functional Image Artifact Correction Heuristic (FIACH) tool for R was used to remove biophysically implausible signal jumps and provide a noise model from signal time courses in brain regions with high noise levels (Tierney et al., 2016). Images were then normalized to a standard MNI space with an isotropic resolution of 2 mm and smoothing was applied using a Gaussian function of 8 mm full width at half-maximum. A second-order Butterworth filter for the fMRI time series was then applied to limit the signal to a low pass frequency of 0.2 Hz, and a high pass frequency of 0.1 Hz, with the signal passed forwards and backwards to avoid phase shifts (Cabral et al., 2017). We also compared the temporal signal-to-noise ratio between our rest, anodal, cathodal and sham conditions confirming no significant differences.

#### Sensorimotor connectivity analysis

**Figure 2:**
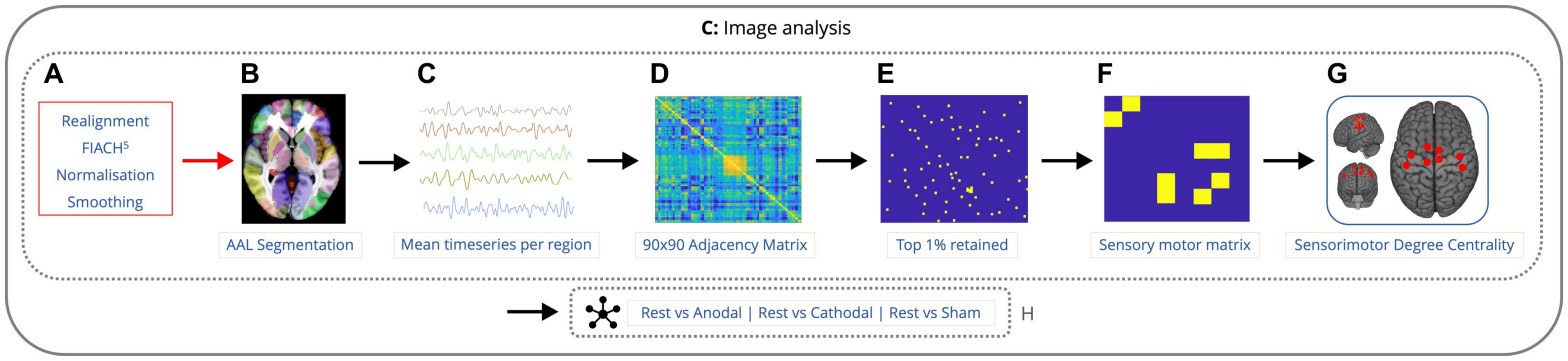
Imaging analysis pipeline showing the order of data processing. Data is preprocessed with realignment, FIACH, normalization to standard space, and smoothing **(A)**. fMRI time-series calculated across voxels in 90 cerebral regions **(B,C)**. 90×90 correlation matrix for each condition, across each run, for every participant **(D)**. Top 1% of the strongest connections were determined and the remaining 99% were omitted **(E)**. Submatrix of the 8 nodes from the 90 by 90 matrix lying in the sensorimotor cortex was created **(F)**. The degree centrality was calculated for each node within the sensorimotor cortex along with the mean degree of connectivity for all the nodes **(G)**. Next, the mean degree of connectivity was computed for the sensorimotor cortex comparing rest to each condition **(H)**.

The mean denoised fMRI time-series was calculated across the voxels in each of the 90 cerebral regions in the Automated Anatomical Labeling (AAL) atlas (Tzourio-Mazoyer et al., 2002). This time series was then partitioned according to the timings of the onset of each condition. A session-specific regressor (consisting of ones and zeros) was included to account for any difference in mean signal between rest epochs. For each condition, across each run, for every participant, whole brain connectivity was assessed using Pearson’s correlation coefficient to generate a 90 by 90 adjacency matrix. The top 1% of the strongest connections for the whole adjacency matrix were determined and the remaining 99% were omitted. A submatrix of the nodes from the 90 by 90 matrix lying in the sensorimotor cortex was created, using the same regions from previous research (Tangwiriyasakul et al., 2019) specifically the primary motor area (left and right), supplementary motor area (left and right), mid-cingulum (left and right), postcentral gyrus (left and right). The degree centrality was calculated for each node within the sensorimotor cortex using the Brain Connectivity Toolbox (Rubinov and Sporns, 2010) and the mean degree of connectivity was calculated across all the nodes. Next, the mean degree of connectivity was computed for the sensorimotor cortex across each of the conditions and runs for each participant (Figure 2). This was performed to provide a single index of local motor network connectivity (Zuo et al., 2012). Degree centrality has been used before in genetic generalized epilepsies as a way to measure alterations in functional connectivity (Wang et al., 2017; Tangwiriyasakul et al., 2018).

#### Statistical analysis

The mean degree of connectivity per run was not normally distributed based on the results of the Shapiro–Wilk Test (*W* = 0.93, *p* = 0.0002). Therefore, a non-parametric statistical test, the Friedman’s ANOVA, was used to compare the mean degree of connectivity of the rest condition to that of the anodal, cathodal, and sham stimulation conditions. To correct for multiple comparisons, a Dunn’s test was applied. Statistics were performed using Prism 9.5.0 (Dotmatics, GraphPad Software, Boston, USA).

## Results

### Feasibility assessment

TES-fMRI data of six healthy controls was included in this study, the mean age was 30.5 years (±7.87 years), and 4/6 were female. The tES paradigm lasted approximately 1.5 h and was well tolerated in both healthy controls and patients, only one scan had to be briefly interrupted due to participant anxiety but could afterwards be completed. No serious adverse events were encountered. This includes seizure induction, cognitive changes, or allergic reactions. Additionally, skin irritation, headaches, nausea or allergic reactions were also not reported by participants for anodal and cathodal stimulation. Healthy participants reported a tingling sensation on their scalp for anodal and cathodal stimulation, but no pain or dizziness. During tACS, all healthy participants reported phosphenes in their visual field. Phosphenes stopped completely when tACS was stopped, but because phosphenes could plausibly induce seizures in patients with photosensitive epilepsy, this condition was not applied to patients. One healthy control experienced a feeling of panic during the first stimulation condition, after being immediately removed from the scanner they were able to re-enter and finish the paradigm without further incident. Two of the three patients had received a routine EEG prior to tES. This was reviewed by a neurologist trained in EEG interpretation (MS). No epileptiform discharges were detected in patients before the tES-MRI.

### Sensorimotor connectivity

**Figure 3:**
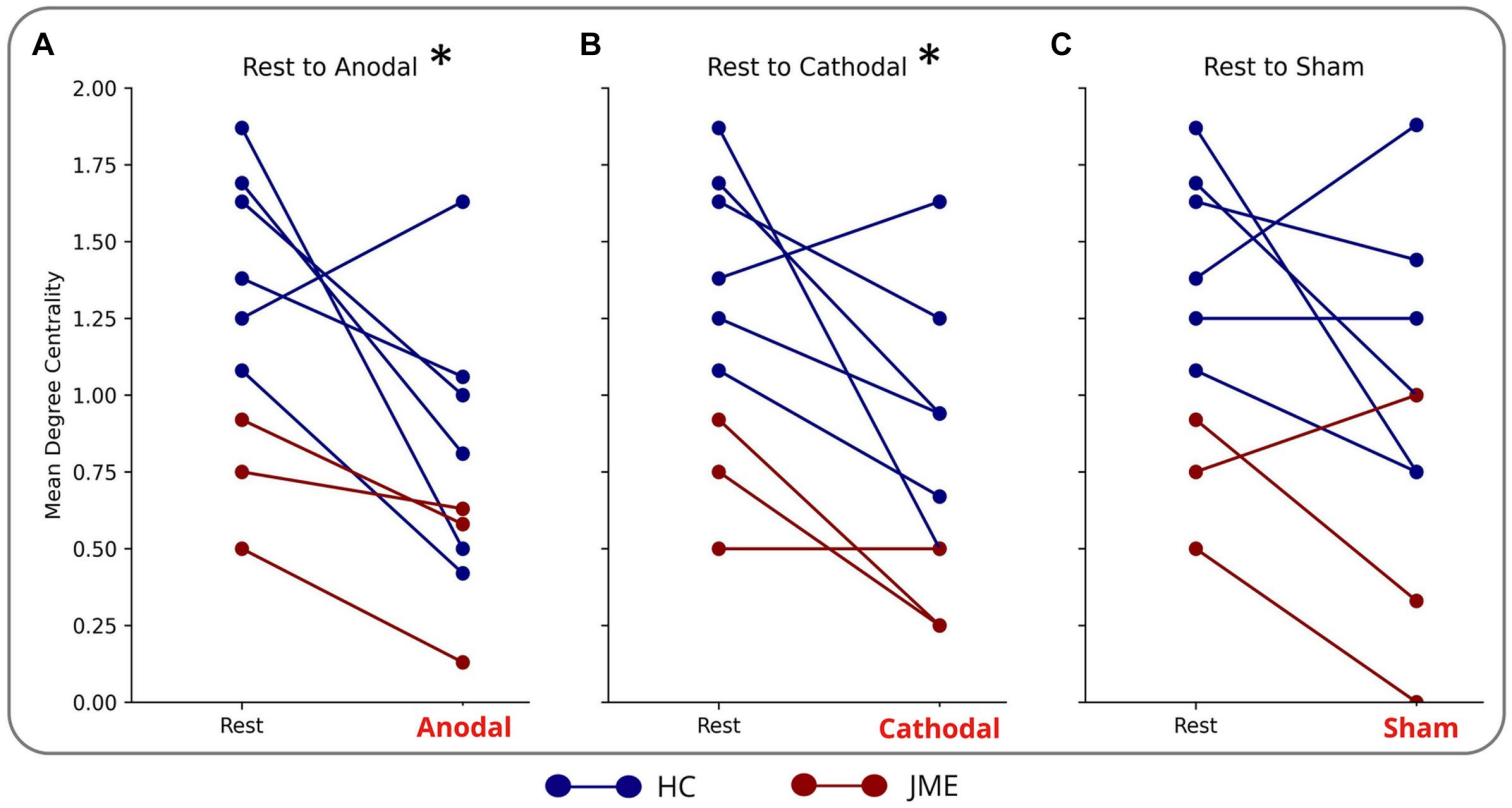
Reduction in mean degree centrality of sensorimotor nodes during **(A)** anodal **(B)** cathodal and **(C)** sham tDCS. Each blue line is a different HC. Each red line is a different patient with JME. *indicates significance: adjusted (*p* = 0.02 for anodal and *p* = 0.03 cathodal).

Anodal stimulation caused a reduction in mean degree centrality of the sensorimotor cortex (comprised of left and right precentral, postcentral, supplementary motor area and cingulum) in 8/9 subjects and cathodal stimulation had the same effect in 7/9 subjects. This culminated in anodal and cathodal stimulation reducing the mean degree centrality of the nodes of the sensorimotor network compared to rest (adjusted *p* = 0.02 and *p* = 0.03 respectively) (Figures 3A,B). There was not a statistically different mean degree centrality of the nodes of the sensorimotor network between rest and sham (Figure 3C).

## Discussion

We have met our primary objective regarding feasibility and tolerability: Anodal and cathodal tDCS were applied in healthy controls and three patients with GGE during fMRI without adverse events and were well tolerated by the subjects. In contrast to our results, a case study suggested a potential health risk of using tES in patients with GGE (San-Juan et al., 2016). We have found that phosphenes were routinely reported during tACS in our healthy controls. Computational head models of similar montages to the one used in our study have been reported in the literature and shown that the electric fields pass through the eye (Iacono et al., 2015). The electrode in the supraorbital area in our montage was close enough to the eye to allow for current to reach the retinas and induce phosphenes. Even montages with electrodes placed only on the occipital cortices are known to induce phosphenes (Lorenz et al., 2019). As explained in the Methods section we decided against using tACS in our patient group due to the potential health risks. Although tACS was not applied in the patient group in this study, owing to the potential risk of inducing seizures in photosensitive epilepsy patients, it has been shown to be an effective means to alter connectivity (Lang et al., 2019; Klink et al., 2020). In this context further investigation of tACS for this purpose should be considered further, utilizing a stimulation montage that can better target the motor network while avoiding stimulation of the visual cortex. Establishing the feasibility of applying tES simultaneously with fMRI in patients with GGE enables the investigation of changes in network connectivity caused by tDCS. This could have a potential therapeutic impact since network changes have been shown to reduce markers of epileptogenicity (Simula et al., 2022). At the same time data from recent years has strengthened the hypothesis that epilepsy is a network disorder (Bartolomei et al., 2017). tES has demonstrated the potential to reduce IEDs and seizures in patients with drug-resistant epilepsy through modulating large-scale brain networks (Simula et al., 2022). In our small sample, no IEDs were present in the EEG recordings prior to tDCS.

Regarding our secondary objective, we have shown that all healthy controls and patients had a significant decrease in degree centrality through anodal and cathodal tDCS. Patients with GGE had an overall lower degree centrality in the sensorimotor cortex than controls, though this could not be statistically assessed in such a small sample, and may be confounded by inter-scanner variability. Two recent systematic reviews on the use of tES in epilepsy reported only one case report in patients with GGE (Sudbrack-Oliveira et al., 2021), it is therefore difficult to compare our findings to preexisting literature. Studies using tES in other types of epilepsy with diffuse onset like Lennox–Gastaut or Rassmussen encephalitis have shown a significant seizure reduction (Auvichayapat et al., 2013; Tekturk et al., 2016). Currently, available studies using tES in epilepsy are overall highly heterogeneous regarding sample characteristics and methodology. For this reason, it is argued that conducting a meta-analysis would create biased effect sizes and estimations (Sudbrack-Oliveira et al., 2021), to date no meta-analysis exists.

Studies investigating tES during fMRI on brain networks of healthy controls lack consensus regarding its efficacy in modulating network function (Ghobadi-Azbari et al., 2021). Looking at individual studies, one study targeting the sensorimotor network in healthy controls through applying cathodal tDCS of 1 mA for 5 min, using the same montage as in our study, resulted in decreased activation in the sensorimotor cortex (Baudewig et al., 2001), concurring with our finding. A study investigating numerous brain networks including the sensorimotor network after the application of 5-min stimulation periods at 2 mA, found connectivity near the applied field and also with remote nodes decreased during tDCS (Leaver et al., 2022). In our research, a similar finding was achieved in patients despite potential differences owing to pathology and medication. This demonstrates network modulation with tES is feasible. Conversely, it has been shown that stimulation for 20 s at 1 mA did not produce a detectable BOLD signal change (Antal et al., 2011). These variable results can among other factors be explained by differences in anatomy (i.e., scalp and skull thickness), placement of electrodes and current intensity (Liu et al., 2018) and analysis approaches. The network effects of tDCS are also dependent on brain state, with cathodal tDCS having greater effects during a task while anodal tDCS has greater effects during rest (Li et al., 2019). Epileptic brain activity, both seizures and IEDs, are often more prevalent during certain states of arousal such as sleep in both focal epilepsy and in GGE (Bernard et al., 2023). This shows that the probability of epileptic activity is modulated by the global state of the brain which relates to cortical excitability.

There is evidence for significant clinical benefit in GGE from the use of VNS and DBS (Haneef and Skrehot, 2023), but a downside of these techniques is their invasiveness, both needing surgery, making non-invasive approaches like tES attractive alternatives if efficacy can be established. Additionally, electric stimulation-driven, non-invasive approaches such as temporal interference have also been shown to reduce epileptiform activity in mouse models and it would be beneficial to analyse how temporal interference affects sensorimotor connectivity with a paradigm like ours (Acerbo et al., 2022). Our preliminary evidence of reduced mean degree centrality of the sensorimotor network supports previous literature about the modulatory effect of tES on the brain. Further confirming our hypothesis, the sham condition was not significantly different from the rest condition and cathodal stimulation significantly reduced the mean degree centrality of the nodes of the sensorimotor network, indicating reduced excitability. In line with previous studies anodal tDCS showed the same results as cathodal tDCS in reducing synchrony (Li et al., 2019; Kurtin et al., 2021).

One key limitation of our research is the sample size of participants. Recruiting patients with JME was cut short by the global COVID-19 pandemic, though the effects of tES on this small group were still powerful enough to produce statistically significant results. While there is a statistically significant difference between the stimulation conditions, a larger group size would add further power and validity to these findings. A further limitation is that the healthy controls were scanned in a different location to the patients, though both were scanned at 3 T. The small sample size and different scanner types precluded conducting a meaningful group comparison because the differences in baseline might be due to either scanner or population. Parameters were matched, however, and the same overall main trend of reduced sensorimotor cortex connectivity was observed within subjects between stimulation conditions in both healthy controls and patients which is not affected by scanner type. Another potential limitation is the effect on SNR. TES has been shown to affect image quality only minimally, with a minor effect on image SNR (Antal et al., 2011). One further limitation is the intake of different anti-seizure medications (ASM) by the patients. Due to the small number included here we could not perform a statistical analysis to account for possible pharmacological effects. Again, although this factor might change overall network connectivity in individuals, the directional reduction in sensorimotor degree centrality between conditions is likely to exist regardless of medication.

## Conclusion

This study provides initial evidence that tES can be safely applied during fMRI in patients with JME. Here, we have also demonstrated sensorimotor network alterations in mean degree centrality that was used as a measure of network connectivity related to overall network synchrony. This preliminary finding appeared to be unrelated to the polarity of the applied stimulation. Further work is required to determine the reliability of this finding in a larger cohort, understand the interaction between current distribution and individual brain structures and establish if the modulation of motor network synchrony can modulate epileptogenicity.

## Data availability statement

The raw data supporting the conclusions of this article will be made available by the authors, without undue reservation.

## Ethics statement

The studies involving humans were approved by London – West London and GTAC & Health Research Authority and Health and Care Research Wales (HCRW): REC reference: 19/LO/1668. The studies were conducted in accordance with the local legislation and institutional requirements. The participants provided their written informed consent to participate in this study.

## Author contributions

ZC: Formal analysis, Writing – original draft. MS: Data curation, Formal analysis, Funding acquisition, Investigation, Methodology, Project administration, Writing – original draft. RP: Formal analysis, Writing – review & editing. CT: Writing – review & editing. MR: Conceptualization, Writing – review & editing. DS: Writing – review & editing. IV: Conceptualization, Data curation, Funding acquisition, Methodology, Project administration, Writing – review & editing. DC: Conceptualization, Data curation, Formal analysis, Funding acquisition, Investigation, Methodology, Project administration, Writing – review & editing.
